# On Inactivation of the Coronavirus Main Protease

**DOI:** 10.1021/acs.jcim.3c01518

**Published:** 2024-02-29

**Authors:** Hong Ha Nguyen, Jim Tufts, David D. L. Minh

**Affiliations:** Department of Chemistry, Illinois Institute of Technology, Chicago, Illinois 60616, United States

## Abstract

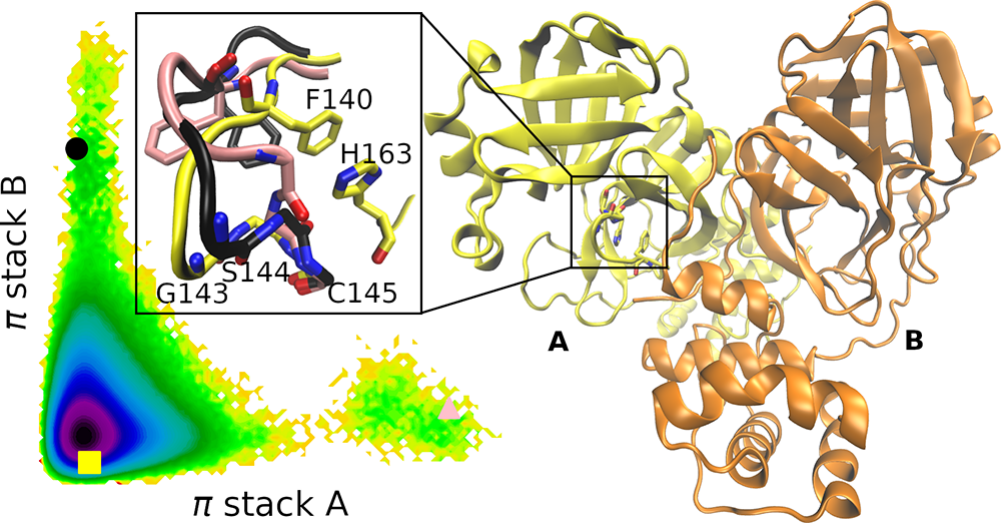

A deeper understanding of the inactive conformations of the coronavirus main protease (MPro) could inform the design of allosteric drugs. Based on extensive molecular dynamics simulations, we built a Markov State Model to investigate structural changes that can inactivate the SARS-CoV-2 MPro. In a subset of structures, one subunit of the homodimer assumes an inactive conformation that resembles an inactive crystal structure. However, contradicting the widely held half-of-sites activity hypothesis, the most populated enzyme structures have two active subunits. We then used transition path theory (TPT) and the Jensen–Shannon Divergence (JSD) to pinpoint residues involved in the inactivation process. A π stack between Phe140 and His163 is a key feature that can distinguish active and inactive conformations of MPro. Each subunit has unique inactive conformations stabilized by π stacking interactions involving residues Phe140, Tyr118, His163, and His172, a hydrogen bonding network centered around His163 and His172, and a modified network of interactions in the dimer interface. The importance of these residues in maintaining an active structure explains the sensitivity of enzymatic activity to site-directed mutagenesis.

## Introduction

The main protease (MPro), also known as the 3C-like or 3CL protease, is an important drug target for coronaviruses including the severe acute respiratory syndrome coronavirus 2 (SARS-CoV-2).^1−4^ MPro is an enzyme that plays a vital role in the viral life cycle, cleaving viral polyproteins at 11 distinct locations to produce shorter, nonstructural proteins that are essential to replication.^1^ It is the target of the Pfizer drug Paxlovid (Nirmatrelvir/Ritonavir), the first oral COVID-19 antiviral approved by the Food and Drug Administration.^5^ While sequence conservation varies across coronaviruses—MPro from SARS-CoV-2 has a sequence identity of 96% with SARS-CoV but only ∼50% with Middle East Respiratory Syndrome^2^—crystal structures show high structural similarity across these coronaviruses,^2^ suggesting that well-designed drugs targeting MPro could work as pan-coronavirus medications.^6,7^ In addition to treating known coronaviruses, such drugs could help control future outbreaks.

A vulnerability of MPro that may be exploited in drug design is its sensitivity to factors including dimerization,^8−11^ ligand binding,^12−15^ and pH.^16,17^ Compared to the homodimer, the MPro monomer has negligible enzymatic activity.^8−11^ Binding of substrates or inhibitors can stabilize the dimer^11,18−21^ and, as suggested by the Hill slope in the concentration response curves of many different inhibitors,^12−15^ possibly alter the binding affinity of the other site. The enzymatic rate constant is also sensitive to pH, dropping dramatically as the pH is reduced below 7.0.^17,22^

**Figure 1:**
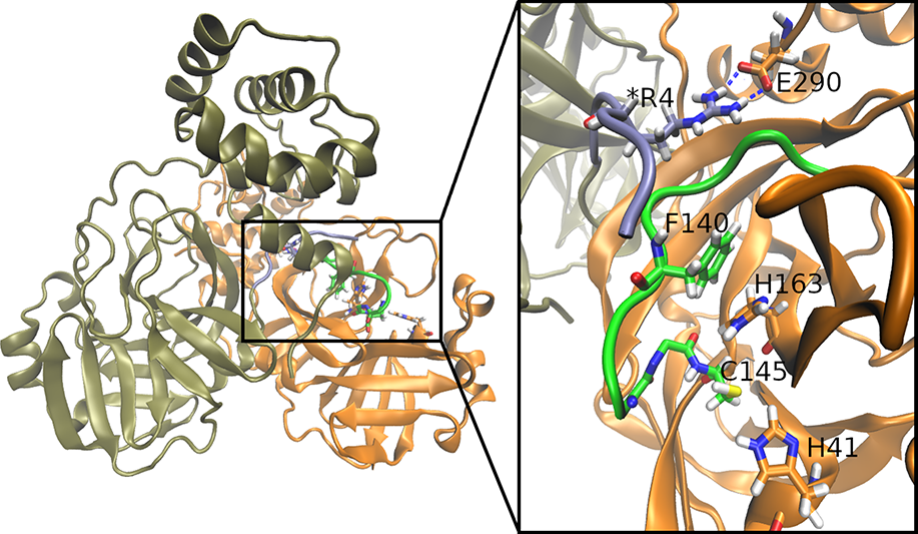
Crystal structure of the SARS-CoV-2 main protease (Protein Data Bank entry 6Y2E^34^) representing the secondary structure with a cartoon with a different color for each subunit. The inset focuses on the area between the dimer interface and catalytic site, which includes the substrate-binding S1 pocket^16^—the catalytic dyad C145 and H41, a π stack between F140 and H163, and a salt bridge between E290 and R4 of the opposite subunit (blue dash line). The side chains of these key residues are shown with a licorice representation. The tubes representing key residues are green for loop L1 (residues 136–145), and iceblue for the N terminus of the opposite dimer. The oxyanion hole, which includes Gly143, Ser144, and Cys145, is depicted with their backbone displayed in a licorice representation.

Regulation of MPro by these factors is driven by conformational changes. Dimerization is important for MPro activity because the N terminus of one subunit stabilizes the active conformation of the opposite subunit (Figure 1).^23−27^ In a crystal structure of MPro at pH 6.0, one of the subunits is in an inactive conformation where the oxyanion hole in the catalytic site (residues 143 to 145, yellow cartoon in Figure 1) is collapsed in comparison to the opposite site and to higher-pH structures.^22^ Constant pH molecular dynamics simulations predict that both the catalytic His41 and His172—which supports the oxyanion hole—have a p*K*_a_ near 6.6 and that protonation of the latter collapses the site.^28^

Improved understanding of inactive MPro conformations can inform the design of allosteric drugs. The catalytic site is not the only region of MPro capable of binding ligands. Simulations show that MPro has multiple cryptic pockets^29,30^ and crystal structures have shown small-molecule fragments binding to allosteric sites.^14,31^ However, binding is not equivalent to inhibition; allosteric drugs must alter the enzyme activity by stabilizing inactive opposed to active conformations of the enzyme. Alternatively, we hypothesize that drugs may be designed to competitively bind to the catalytic site of one subunit while stabilizing the inactive conformation of the opposite subunit and disabling the entire protein.

Here, we analyze the transition between active and inactive conformations observed in extensive molecular dynamics simulations of the SARS-CoV-2 MPro dimer. The simulations were produced by Folding@Home (2.079 ms of aggregate simulation)^30^ and D.E. Shaw Research (a single 100 μs simulation)^32^ in the early part of the pandemic. We used the Folding@Home simulations to build a Markov State Model (MSM), which describes the kinetics of transitions between conformations.^33^ Second, we applied transition path theory (TPT) to identify the highest-flux paths between the active and inactive states of each subunit in the MSM. Next, we used the Jensen–Shannon Divergence (JSD) of the dihedral angles between TPT states to identify residues that play the most essential roles in this symmetry-breaking transition. Finally, we compared the proposed mechanism with the series of transitions observed in the single long trajectory by D.E. Shaw Research, which was produced by using a different force field.

## Methods

### MSM Construction

MSMs are a complete description of the microstates of a system, the kinetics of transitions between them, and their equilibrium populations.^33,35^

We built a MSM based on molecular dynamics trajectories of the apo (no ligand) MPro dimer that were produced by Folding@Home.^30^ In both the Folding@Home and DESRES simulations, all histidine residues were neutral and in the same tautomeric form (Table S1). Trajectories from PROJ14234, PROJ14542, and PROJ14543 were downloaded from the MolSSI/BioExcel COVID-19 Hub^36^ under “Folding@home simulations of nsp5 (2.9 ms)”. PROJ14584 was unavailable for download. The Trjconv utility in GROMACS^37^ 2021 was used to unwrap periodic boundary conditions. Even after unwrapping, the dimer appeared dissociated in some frames. A salt bridge (Glu290*-Arg4) connecting the N-terminal domain I to domain III of the opposite subunit is pivotal for the dimerization of MPro (Figure 1).^19,38^ Thus, frames were considered unfixable when the minimum distance between either OE1 or OE2 of Glu290 and either NH1 or NH2 of Arg4 of the opposite subunit was larger than 18 Å after the unwrapping operation. While such observations could indicate dissociation, we observed that they occurred in sporadic spurts of several picoseconds as opposed to gradually growing over longer time periods and treated them as artifacts of the unwrapping process. To maintain the continuity of trajectories, unfixable frames and all subsequent frames were also removed. The total simulation time used for the analysis was 2.079 ms.

We developed a procedure for categorizing configurations that separate active and inactive configurations of MPro into different microstates. Building an MSM requires a procedure for categorizing configurations, which exist in a continuous space, into discrete microstates. The categorization is typically performed by clustering. Initially, we used a standard procedure^39^ that we will refer to single-layer clustering. First, time-lagged independent component analysis (tICA)^35^ was performed on backbone torsions and side chain χ1 and χ2 angles using PyEMMA^39^ 2.5.12. Next, *k*-means clustering was performed based on the Euclidean distance between all the dimensions of the tICA using scikit-learn^40^ 1.2.0. This standard procedure is intended to separate configurations separated by slow transitions into distinct microstates. Unfortunately, the procedure led to microstates that included both active and inactive conformations. Evidently, the transition between active and inactive conformations was not among the slowest processes of the molecular dynamics. Consequently, we developed a two-layer clustering approach. The purpose of the first clustering layer was to differentiate between active and inactive conformations. In the second layer, clustering was based on the standard procedure.

To decide how to perform the first layer of clustering, we considered requirements that Chen et al.^8^ compiled for SARS-CoV MPro enzyme activity: a suitable oxyanion hole, Tyr-Xaa-His motif, hydrophobic packing between Phe140 and His163, and the conformation around Glu166. Chen et al.^8^ defined a good oxyanion hole based on distances between the Gln residue of the substrate and the amide groups of Gly143, Ser144, and Cys145.^8,41^ As the simulations we analyzed lacked a substrate, we instead estimated pairwise distances between the amide groups of these three residues. All other features were based on the described distances. For each feature, we defined an activity fingerprint, a vector with a value of 1 for each structure with distances less than a threshold and 0 for larger distances. Thresholds were determined by identifying peaks in the free energy landscapes (Figure S1), except for the distances between Glu166 and His172, which were fixed at 4.5 Å; no hydrogen bond can exist at this distance. The energy landscapes of each feature were from single-layer clustering. Activity fingerprints were computed for every configuration in the Folding@Home trajectory. Feature vectors were compared using the Tanimoto coefficient.^42^ Based on our analysis (described in the results section), we performed the first layer of clustering based on the distance (in Å) between the center of mass of the aromatic rings in Phe140 and His163 for each subunit.

Active and inactive conformations were defined by clustering using these pairs of distances. *k*-means clustering as implemented in scikit-learn^40^ 1.2.0 was performed with three centers chosen as *d*_A_ = 4 and *d*_B_ = 4 for active–active, *d*_A_ = 4 and *d*_B_ = 10 for active–inactive, and *d*_A_ = 10 and *d*_B_ = 4 for inactive–active, where *d*_A_ and *d*_B_ refer to Phe140-His163 distances (in Å) in subunit A and B, respectively. We considered the distance of 4 Å to be active because active crystallographic structures have comparable distances; Protein Data Bank entry 6Y2E^34^ has distances of 3.82 and 3.96 Å. Subsequently, we will use a pair of numbers separated by a dashed line to denote whether the subunits are active–active (1–1), active–inactive (1–0), or inactive–active (0–1), where the first number refers to subunit A and the second to subunit B.

The second layer of clustering, intended to separate kinetically distinct microstates of the system, was performed by using a standard MSM procedure. *Within* each of the three larger clusters, *k*-means clustering was performed based on Euclidean distances between the tICA described above. The number of clusters selected for the 1–0 and 0–1 states was 10 and for the 1–1 state was 2480. Thus, our MSMs have 2500 microstates, as in Zimmerman et al.^30^

After defining these microstates, we selected a lag time and estimated a transition probability matrix for the MSM. Implied time scales represent the relaxation time scales of a system, as inferred by a MSM transition matrix estimated at a specific lag time (τ). Relaxation time scales are an inherent physical property of the simulated system and are expected to be independent of the lag time. The selected lag time must be long enough for the implied time scales to converge; if the lag time is too short, then the relaxation time scale will also be too short. Based on the trend of the implied time scale as a function of lag time (Figure S2), we chose a lag time of 4.0 ns. Transition probability matrices were then estimated as in Zimmerman et al.^43^—counting transitions between microstates, adding 1/*n* to the count (where n is the number of states), and normalizing—as implemented in Enspara.^44^ Equilibrium populations were calculated as eigenvalues of the obtained transition probability matrix.

### Binding Site Analysis

To facilitate binding site analysis, we wrote a new Python package, Occupancy Fingerprinter, which efficiently computes occupancy fingerprints.^45^ An occupancy fingerprint is a grid-based binary featurization of protein structures or substructures based on the van der Waals (vdW) volume that the structure occupies. The grid is defined at evenly spaced points in a cubic grid. If there is an atom within a vdW radius of a grid point, then the corresponding value of the occupancy fingerprint is one. Only atoms within a spherical region with a specified center coordinate, radius, and grid spacing are considered. Otherwise, it is zero. Previously, we computed occupancy fingerprints using a variation of POVME.^46,47^ We optimized Occupancy Fingerprinter by changing the loop structure and by translating it to Cython,^48^ a typed and compiled version of Python. The program computes occupancy fingerprints by initializing all points to zero and iterating over all atoms, setting any grid point that falls within the vdW radius to one. This approach is faster than iterating over all grid points and looking for nearby atoms. Other new features of the package are that it leverages multiprocessing and uses the advanced trajectory handling of MDTraj,^49^ allowing it to handle very large data sets in a time-efficient manner. The resulting featurized trajectory is saved in a compressed HDF5 file that can be used for further analysis. Occupancy Fingerprinter is publicly available under the open source MIT license at https://github.com/jimtufts/occupancy_fingerprinter.

We compared the binding sites of simulated and crystallographic structures using the features generated by occupancy fingerprinter.^45^ The crystallographic structures were 803 inhibitor-bound structures solved as part of the COVID Moonshot,^14^ two substrate-bound structures (PDB entries 7T8M and 7T8R),^6^ and an inactive structure (PDB entry 7NIJ).^51^ Grid points with a spacing of 0.5 Å were selected to be within 8 Å of the center of the catalytic sites of both subunits, which were defined as the center of the geometry of residues 141–145 and 164–166. Occupancy fingerprints of both sites were categorized into 75 clusters using *k*-means clustering. A small fraction of these configurations (2.5%) were randomly sampled from each cluster for a total of 519,916 configurations. Principal components analysis, as implemented in pyEMMA^39^ 2.5.12, was applied to the occupancy fingerprints of these configurations as well as the crystallographic structures.

### Coarse-Graining of Transition Paths

TPT is a statistical framework that focuses on the analysis of reactive trajectories between two predefined sets. It quantifies the proportions of a Markov process path during which the trajectory transitions from set A to set B.^52^ We applied, TPT, as implemented in PyEMMA^39^ 2.5.7, to extract the most dominant pathway and their fluxes between the 1–0 and 0–1 states. Applying TPT yields a network composed of many highly branched pathways with corresponding fluxes. To simplify the result, we grouped microstates into intermediate states based on appearing between 1−0 and 0–1 in the six pathways with the highest flux (Table S1), representing over 95% of the total flux. Specifically, state I1 comprises the first microstates accessed after 1–0, with probabilities higher than 1% of the total flux; state I3 includes the last microstates accessed before the selected 0–1 state; state I2 encompasses microstates appearing between states I1 and I3; and state I4 represents all other microstates.

### Jensen–Shannon Divergence

The JSD, a metric quantifying differences between two probability distributions,^53−55^ was utilized to conduct pairwise comparisons of the probability densities associated with the torsion angles of all residues (across both chains) across the five states of the most dominant pathway. We computed backbone ϕ and ψ and side chain χ1 and χ2 torsion angles of each residue were calculated using PyEMMA.^39^ One-dimensional probability densities of each torsion were calculated using Gaussian Kernel density estimation in scipy^56^ 1.5.3 with a bandwidth of 0.05. Each sample was weighted by its equilibrium probability from the MSM. Because torsion angles are periodic, we triplicated each sample at a torsion angle Θ at Θ ± 2π. This ensured that the probability density function at the periodic boundaries π and π are equal. The probability density function was normalized based on the integral between −π and π. The JSD between a pair of probability distributions *P* and *Q* is
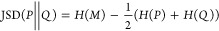
1where . *H*(*P*) is the Shannon entropy,
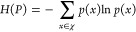
2where *x* is a possible value of a discrete random variable and *p*(*x*) is the probability density of the distribution *P*. The Shannon entropy of each torsional probability density function was estimated for 10,000 evenly spaced angles between −π and π. As there are multiple torsion angles associated with each residue, we used the largest JSD between corresponding torsion angles to represent the JSD between a pair of residues.

## Results

### Simulated Structures Resemble Crystallographic Structures

**Figure 2:**
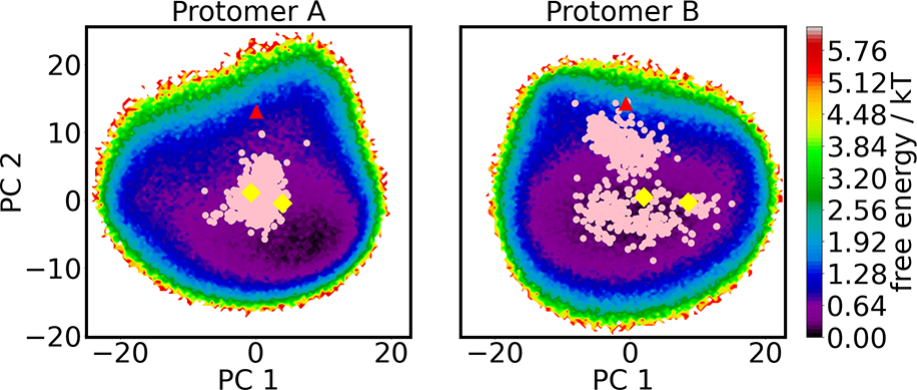
Comparison of MPro binding pocket conformations of each subunit from ∼500 k frames of molecular dynamics simulations (colored energy landscape) and multiple crystal structures: one inactive (red triangles); two substrate-bound (yellow diamonds); and 803 inhibitor-bound (pink circles). The first two projections are shown for principal components analysis performed on the occupancy fingerprints of each monomer. The energy landscape is based on the Markov State Model built using single-layer clustering.

Principal component analysis of occupancy fingerprints shows that catalytic site structures observed in *apo* (unbound) simulations are a superset of *holo* (ligand-bound), substrate-bound, and inactive structures observed in crystallography (Figure 2). This observation confirms that simulated structures are capable of binding ligands and substrate. For many ligands, holo conformations of protomer B are in the ground state of the apo protein. There is another set of ligands for which the holo conformation has a free energy between 0.5 and 2 kT, a barrier than can be readily compensated for by the binding process. For the binding of all ligands to protomer A, the free energy of rearranging the subunit to the holo conformation is less than 1 kT.

While both sets of MD simulations were initiated from symmetric crystallographic structures in active conformations (PDB entry 6Y84 for DESRES and PDB entry 6Y2E for Folding@Home), they both access conformations that resemble inactive crystallographic structures (Figure S3). The shorter DESRES simulation features a more localized flip of F140 away from the catalytic dyad (purple structure in Figure S3b), resembling an inactive crystallographic structure of SARS-CoV-1 MPro (green structure from PDB entry 1UJ1 in Figure S3a) that was used to introduce the concept of an inactive MPro structure.^16^ In the longer Folding@Home simulations, a larger rearrangement of the oxyanion hole is observed (pink structure in Figure S3b); as in an inactive crystallographic conformation of SARS-CoV-2 MPro^51^ (orange structure from PDB entry 7NIJ in Figure S3a), the oxyanion hole comprising Gly143, Ser144, and Cys145 (Figure S3a) collapses near His163. While these inactive structures all differ in the exact orientation of Phe140, the similarity of the oxyanion loop between the simulated and crystallographic structures provides experimental evidence that the simulations are realistic.

### Most Features of Active Conformations Occur Concurrently

**Figure 3:**
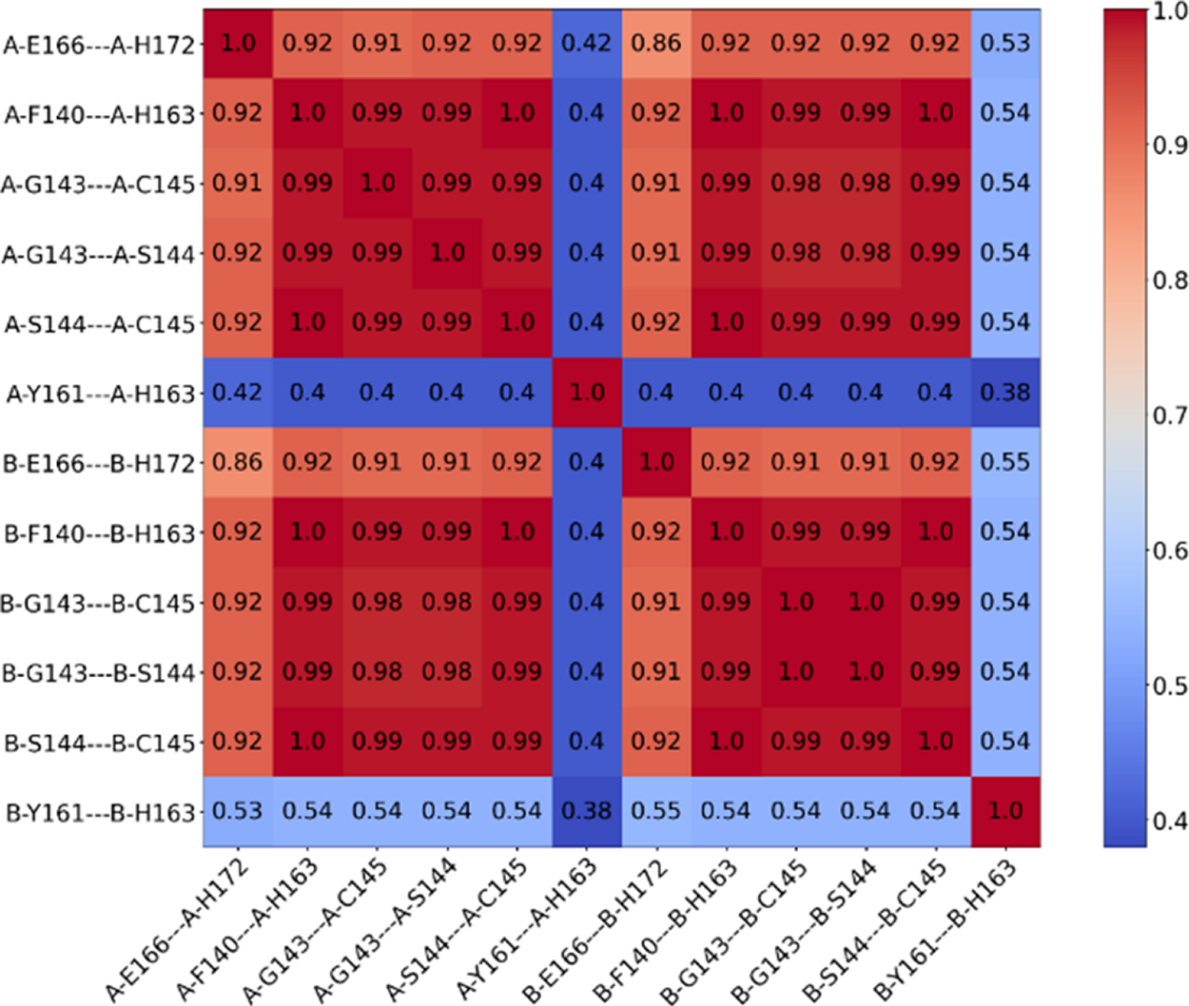
Tanimoto coefficients between activity fingerprints of catalytic site features crucial for MPro activity. Six distances between Phe140 and His163, Gly143 and Ser144, Ser144 and Cys145, Glu166 and His172, and Tyr161 and His163 in both subunits were calculated, resulting in a total of 12 features.

To help us identify a feature capable of distinguishing active and inactive conformations, we computed activity fingerprints for all structures in the Folding@Home trajectories. Tanimoto coefficients between the fingerprints range from zero for completely distinct fingerprints to one for identical fingerprints. For our activity fingerprints, Tanimoto coefficients show that nearly all tested features have distances that are higher or lower than the threshold in the same structures (Figure 3). For the hydrophobic packing between Phe140 and His163 and the oxyanion loop (represented by distances between Gly143, Ser144, and Cys145), Tanimoto coefficients are nearly one. This suggests that breaking the π stack between Phe140 and His163 is tightly associated with the collapse of the oxyanion hole. Tanimoto coefficients between these features and the Glu166-His172 distance are lower, about 0.9, but still near one. In contrast, the activity fingerprints for the Tyr161-His163 distance have a much lower Tanimoto coefficient; the conformation of the Tyr-Xaa-His motif is relatively independent of the other tested features. These results show that the Phe140-His163 distance is a reasonable proxy for the conformation of the oxyanion hole, justifying its use to categorize structures as active or inactive.

### Two-Layer Clustering Provides Similar Kinetics as Single-Layer Clustering

We validated our novel two-layer clustering approach by comparing kinetic properties with the single-layer *k-*means clustering of the tICA. We observe that the two clustering approaches lead to similar convergence of the implied time scales (Figure S2 in the SI.) Moreover, the VAMP-2 score—a metric from the variational approach for Markov processes^57^ that quantifies how accurately a time series is modeled by a Markov model—are essentially indistinguishable: 9.92499 for clustering based on tICA and 9.91393 for two-layer clustering. These results demonstrate that using two-layer clustering does not significantly reduce the quality of kinetic models compared to the standard approach. The benefit of two-layer clustering is that microstates clearly distinguish active and inactive configurations.

### The MPro Dimer Favors Active Conformational States

**Figure 4:**
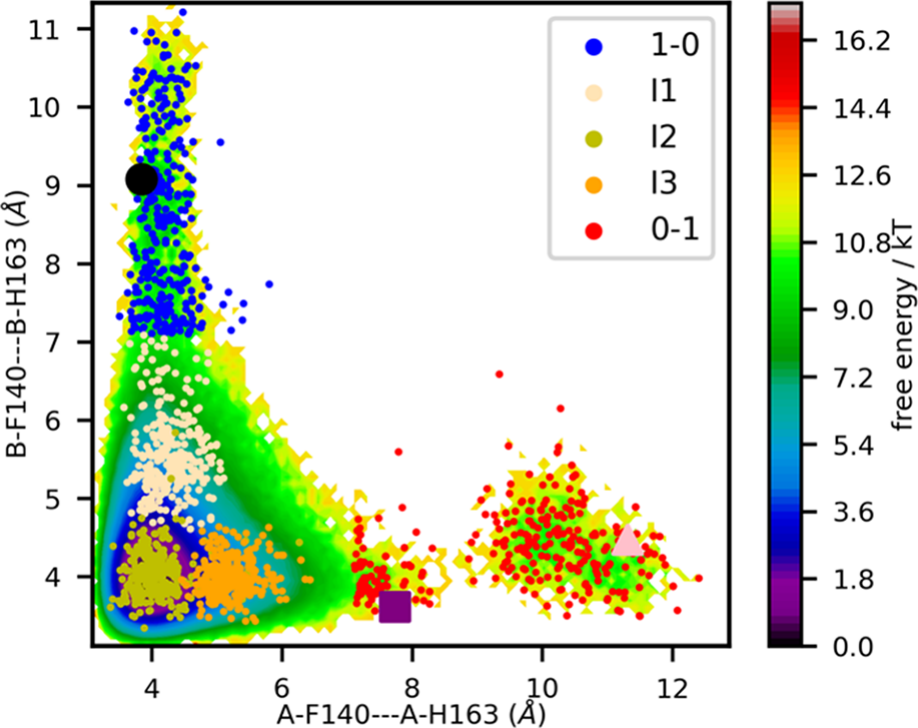
Free energy landscape of the π stack between Phe140 and His163 in subunit A (*x* axis) and subunit B (*y* axis). Free energies are from the MSM built from Folding@Home simulations, using two-layer clustering. Distances are between the centers of mass of the aromatic rings. Colored points are random samples from each microstate in the MSM based on two-layer clustering, colored according to the macrostate membership (1–0, I1, I2, I3, or 0–1) of the most dominant pathway. Macrostates I1, I2, and I3 are subsets of 1–1. Representative structures shown in later figures are denoted by a black circle, which is taken from 1−0, purple square from 0−1, and pink triangle from 0−1. These structures are visualized in other figures using a licorice representation with the corresponding colors.

The minimum of the free energy landscape, which occurs near *d*_A_ = 4 and *d*_B_ = 4 (Figure 4), corresponds to a configuration in which both subunits are active. The free energies of both 1–0 and 0–1 are at least 9.0 kT higher than this minimum. No configurations were observed in which both subunits are inactive. The existence of a crystallographic structure with two inactive subunits demonstrates that such a conformation is sterically feasible.^51^ Thus, the failure to observe such a structure in a long but unbiased MD simulation is due to a force field error or the low joint probability of independently observing two inactive subunits. To the best of our knowledge, no simulations using any force field that were started from active subunits have transitioned to configurations with two inactive subunits. The evaluation of force field error is beyond the scope of the current work.

Another key trait of Figure 4 is that the observed energy landscape is asymmetric. There is a distinct gap between the active and inactive states of subunit A, suggesting a large free energy barrier between the states. In contrast, we observe a continuous series of conformations between the active and the inactive states of subunit B.

### Inactivation Transitions Are More Frequent in Chain B than A

The asymmetry of the energy landscape indicates that the subunits have different mechanisms of inactivation. To investigate these mechanisms more carefully, we applied TPT to transitions between the asymmetric states 1–0 and 0–1. When 1–0 is designated as the reactant and 0–1 as the product, about 50% of the total flux between asymmetric states flows through the most dominant path. The most dominant path involves going down and to the right of Figure S4: down from 1 to 0 to I1 and then to the symmetric state I2 before turning to the right to I3 before reaching 0–1. In addition to this most dominant path, most of the total flux (>95%) can be accounted for in the first six coarse-grained pathways (Table S2). Each of the remaining pathways contributes less than 2% to the total flux.

Beyond key pathways and associated fluxes, TPT also shows that transitions between active and inactive states are more frequent in subunit B than subunit A. Committor probabilities describe the probability that a system in state *i* will reach the product state before returning to the reactant state.^58^ The committor probability for I1 is 0.343. It increases gradually for the intermediate states I2 (0.347), I3 (0.352), and I4 (0.353) but remains below 50% for I4; for all intermediate states, it is more probable to return to 1–0 than to proceed to 0–1. The relatively low probability of transitioning between active and inactive states of subunit A is consistent with the gap in the free energy surface observed in Figure 2. For the transition from 0–1 to 1–0, the committor probability is the opposite (one minus the committor probability for the transition from 1–0 to 0–1) and the fluxes are the equal but in the opposite direction (Figure S4).

### Inactivation Involves Local Conformational Changes Including the N Terminus of the Opposite Subunit

**Figure 5:**
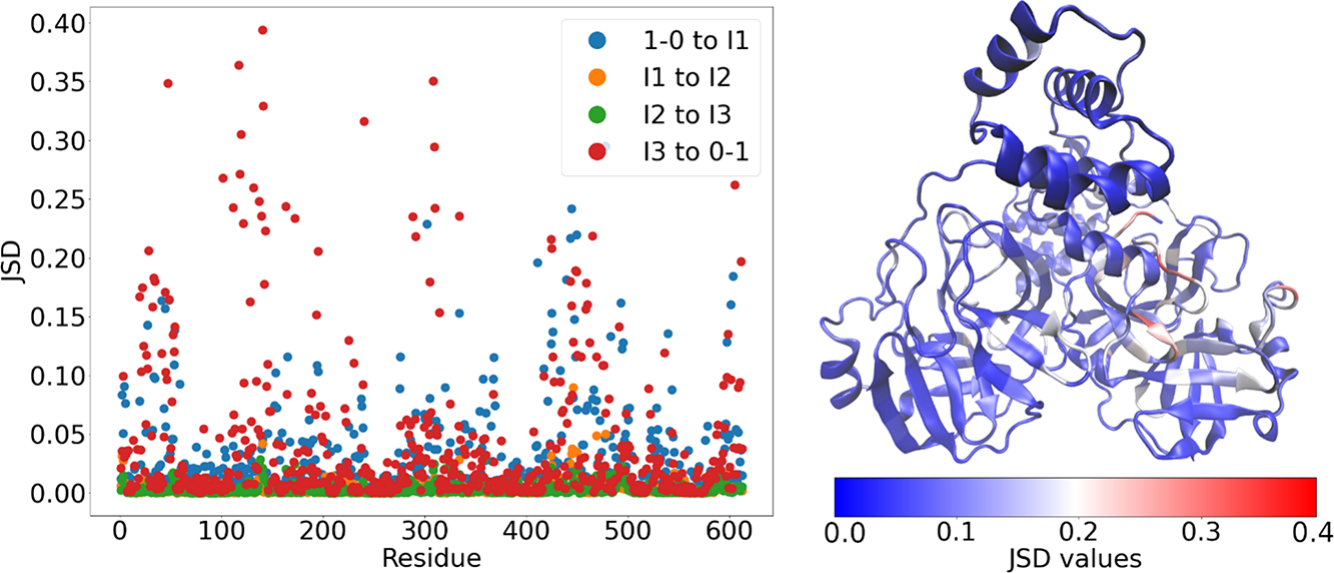
JSD values of the residue torsion angles over transitions. For each residue, the torsional angle with the maximal JSD value was used. Each residue has JSD values for four transitions, from 1–0 to I1, from I1 to I2, from I2 to I3, and from I3 to 0–1, which are colored blue, orange, green and red, respectively. The projection of JSD values onto the crystal structure (PDB entry 6Y2E) on the right side of the figure corresponds to the transition from I3 to 0–1.

JSD values range from zero, indicating identical distributions, to one, indicating no overlap between distributions.^59^ The JSD values demonstrated a significant increase during the transitions between 1−0 and I1, as well as between I3 and 0–1, compared to the other transitions (Figure 5). This finding further supports using the distance between Phe140 and His163 as a criterion to define the active and inactive states of MPro; most conformational changes occur during the transitions between active and inactive states in each subunit. During the inactivation of subunit A, significant conformational changes occur in the region containing the oxyanion hole (residues 143–145), loop L1 (residues 136–145, Figure 1), and specific residues such as Cys117, Tyr118, Asn119, His163, His172, and the N terminus (residues 2–4) of the opposite subunit (Figures 5 and S5c,d). Similarly, the same regions in subunit B also exhibit higher JSD values, although the difference is relatively small compared to subunit A.

In addition to pinpointing regions that show the largest changes upon macrostate transitions, the JSD was also used to identify differences between subunits within each macrostate (Figure S6). In all of the intermediate macrostates (I1, I2, and I3), differences between subunits A and B are relatively small. This shows that the 1–1 states, which we defined purely on the basis of the Phe140-His163 distance, are indeed quite symmetric overall. In contrast, for the asymmetric macrostates (1–0 and 0–1), JSD differences between subunits are comparable in magnitude to JSD differences between macrostates (Figure 5).

Our approach of combining MSM, TPT, and JSD analyses more specifically identifies residues involved in inactivation than crystal structures alone. When comparing active and inactive subunits of crystal structures, many residues exhibit significant torsional changes (Figure S7). Whereas the JSD identifies specific residues mostly close to the catalytic site (Figure 5), residues with large torsional changes are distributed throughout the entire protein and most are unlikely to be involved in inactivation. Many of these changes may be serendipitous or due to crystal contacts.

We focus our subsequent analysis on these residues near the catalytic sites. While there is a possibility that the JSD analysis is incorrect and other more distant residues also play a role in MPro inactivation, the analysis of these residues is beyond the scope of our present work.

### An Inactive Conformation of Subunit A Is Stabilized by a π Stack between Phe140 and Tyr118 and a Hydrogen Bonding Network Involving B-Ser1

**Figure 6:**
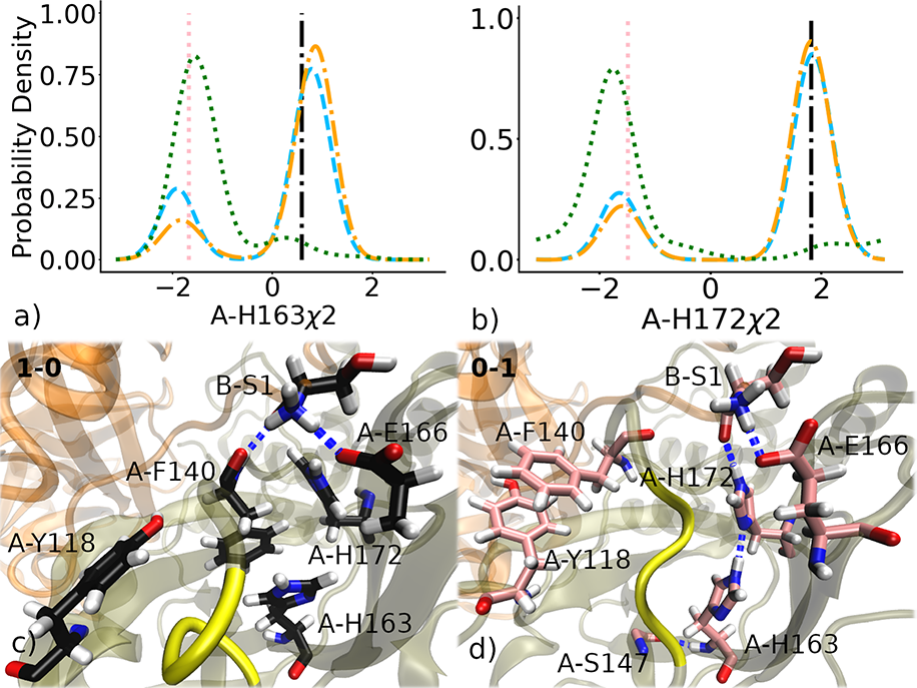
Inactivation of subunit A. (a, b) Probability distributions of the χ2 angles, which specify rotation of the imidazole ring, of His163 and His172 in subunit A for 1–0 (cyan dashed), 1–1 (orange dash-dotted), and 0–1 (green dotted) states. The 1–1 state is a weighted sum of I1 and I3. Angles observed in two of the representative structures (shown as a black circle and pink triangle in Figure 2) are shown as vertical lines of the corresponding colors. (c, d) These two representative structures are shown (coded by the color of the licorice) with the tan cartoon for subunit A and orange for subunit B. Blue dashed lines represent H-bonds.

In active conformations of subunit A, Phe140 is part of a sandwich π stack of His163 and a T-shaped π stack with His172 (Figure 6c). The χ2 angles of A-His163 and A-His172, which correspond to the rotation of the imidazole ring, exhibit bimodal distributions that are shown as orange and blue dashed lines in Figure 6a,b. These distributions show that there are two distinct conformations of the imidazole ring for each histidine residue, related by a 180° flip, which are stabilized by a three-body interaction. In contrast, the χ1 angle of Phe140 and backbone torsion of Tyr118 have a single peak in the active conformations of subunit A (Figure S8). Active conformations are also stabilized by hydrogen bonds between B–S1, the side chain of Glu166, and the backbone of Phe140 (Figure 6c). Upon inactivation of subunit A, the χ2 angles of A-His163 and A-His172 shift to an alternative conformation, which is represented by the pink structure shown in Figure 5d. In this inactive conformation, A-Phe140 rotates away from A-His163, and the rotation is stabilized by a π stack with Tyr118. There are also distinct shifts in the χ1 angle of Phe140 and backbone torsion of Tyr118 (Figure S8). This inactive conformation of His172 and His163 is stabilized by a network of hydrogen bonds between them and neighboring residues in the S1 pocket, including A-Glu166, A-Ser147, and B-Ser1 (Figure 6d). In summary, for subunit A, Phe140 acts as a switch by rotating between two stable π stacking interactions made with either His163 or Tyr118, leading to an energy gap observed in the energy landscape shown in Figure 4.

### An Inactive Conformation of Subunit B Is Stabilized by π Stacking between Phe140 and Tyr118 and between His163 and His172

**Figure 7:**
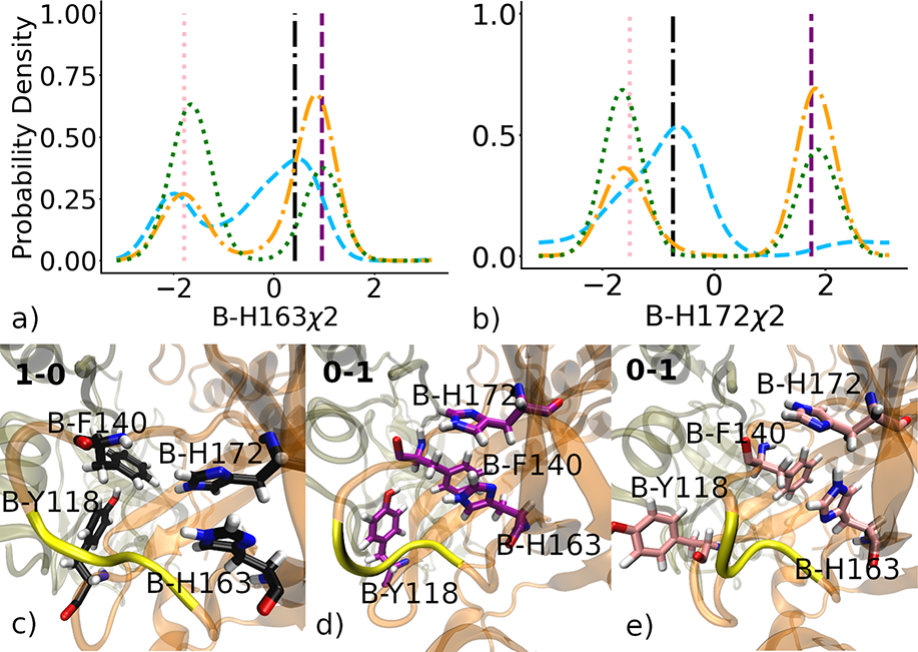
Inactivation of subunit B. (a, b) Probability distributions of the χ2 angles, which specify rotation of the imidazole ring, of His163 and His172 in subunit B for 1–0 (cyan dashed), 1–1 (orange dash-dotted), and 0–1 (green dotted) states. The 1–1 state is a weighted sum of I1 and I3. Angles observed in the three representative structures are shown as vertical lines of the corresponding colors. (c–e) These representative structures are shown (coded by the color of the licorice) with the cartoon colored tan for subunit A and orange for subunit B.

Active conformations of subunit B have the same system of aromatic interactions between Phe140, His163, and His172 (Figure 7d,e) as subunit A. In contrast, the inactive state of subunit B is stabilized by a different set of aromatic interactions: a T-shaped π stack between Phe140 and Tyr118 and a sandwich π stack between His163 and His172 (Figure 7c). As evidenced by the broader distributions of χ2 angles, the latter π stack is less clearly defined than the π stacks in the active states, not only for subunit B but also for the active and inactive states of subunit A (Figure 6a,b). Thus, the distance between aromatic rings in Phe140 and His163 is also more broadly distributed in the inactive state of subunit B than in the inactive state of subunit A, as seen in the energy landscape (Figure 4).

### Communication between Subunits Occurs via the Dimer Interface

**Figure 8:**
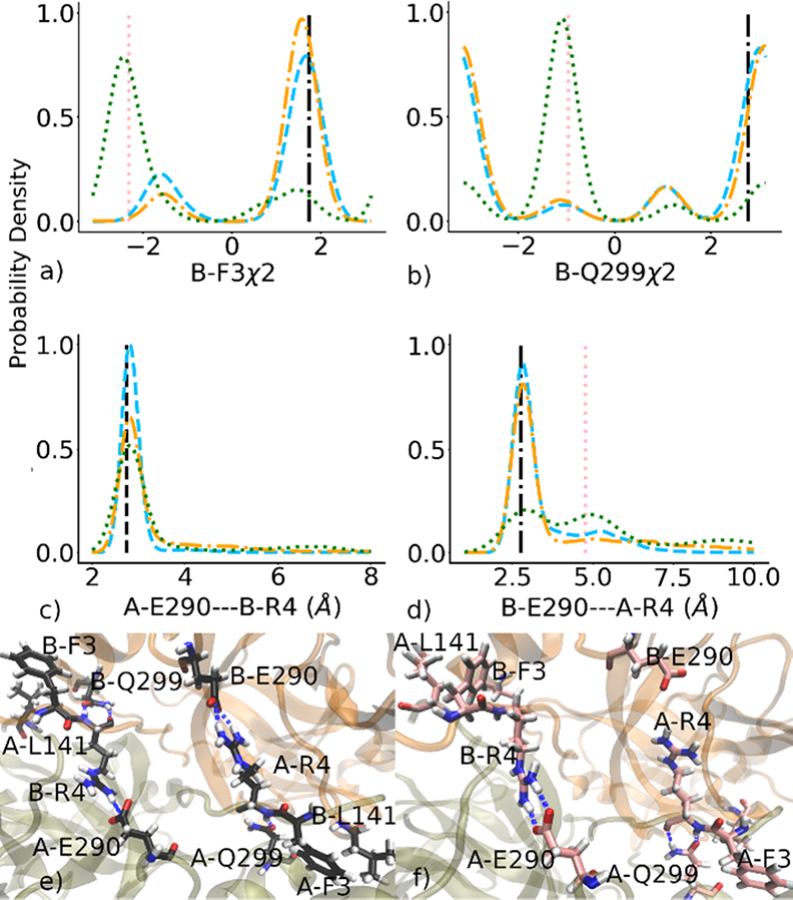
Conformational changes of SARS-CoV-2 MPro subunits from active to inactive are coupled with residues in the dimer interface. (a, b) Probability distribution of the χ2 angles of B-Phe3 and B-Glu299. (c, d) Probability distribution of the distances between A/B-Glu290···B/A-Arg4. Data for the 1–0, 1–1, and 0–1 states are cyan dashed lines, orange dashed-dotted lines, and green dotted lines, respectively. The black and pink vertical lines indicate the corresponding values of representatives taken from the Folding@Home simulation. (e, f) Representatives (black and pink-colored licorice structures) of two main states of the χ2 angles of B-Phe3 and B-Glu299. The subunits A and B are represented by the tan and orange cartoon structure, respectively, and the blue dashed line represents the H-bonds.

Both residues located in the S1 pocket and the N-finger residues (1–7) play a critical role in the dimerization of MPro.^26^ Changes in the dihedral angle distributions of these residues are linked to the transition between the active and inactive conformations in both subunits. When subunit A becomes inactive, there are noticeable changes in the χ2 angles of B-Phe3 and B-Glu299 (Figure 8a,b). These rotations enable the side chains to participate in different sets of interactions. Specifically, when subunit A is inactivated, B-Phe3 forms a long-range hydrophobic interaction with A-Leu141 (Figure S9a,d), and the H-bond between B-Glu299 and B-Arg4 almost disappears (Figure S9b,e–g). Inactivation of subunit A is accompanied by changes in the key salt bridge between the two subunits. Regardless of conformation, the MPro dimer contains a salt bridge between A-Glu290 and B-Arg4 (Figure 8c). On the contrary, when subunit A is rendered inactive, the salt bridge connecting B-Glu290 and A-Arg4 is frequently disrupted (Figure 8d). The hydrophobic interaction involving A-Leu141 and B-Phe3 intensifies the binding between domain III (residues 201–306) of subunit B and domain I (residues 1–101) as well as domain II (residues 102–184) of subunit A, inducing strain on the salt bridge on the opposite side (B-Glu290 and A-Arg4) and thus weakening it. This phenomenon is exclusively manifested in subunit A, contributing to the observed asymmetrical energy landscape depicted in Figure 4.

Representative conformations, marked by the black circle, purple square, and pink triangle in Figure 4, provide further insight into MPro dimerization. In 1–0 and 0–1 states, represented by the black and purple structures, respectively, A-Phe140 predominantly engages in hydrophobic interactions with B-Met6 and A-Tyr126 (Figure S9c). However, in the fully inactive 0–1 state, represented by the pink structure, A-Phe140 undergoes a rotation of its χ1 angle to more populated states (Figure S8a), and its backbone bends, enabling it to interact aromatically with A-Tyr118 (Figure S8d). Meanwhile, A-Leu141 is optimally positioned for hydrophobic interaction with B-Phe3 (Figure S9d). This transition from a less-populated state to a more populated one necessitates a high-energy activation, signifying the formation of hydrophobic interactions between domain III of subunit B and domain II of subunit A in the dimer interface, leading to the generation of more stable inactive conformations of subunit A. The asymmetrical communication between the two subunits explains why inactive states of subunit A are less favored than those of subunit B.

### DESRES MD Simulation Provides an Example Sequence of MPro Inactivation

**Figure 9:**
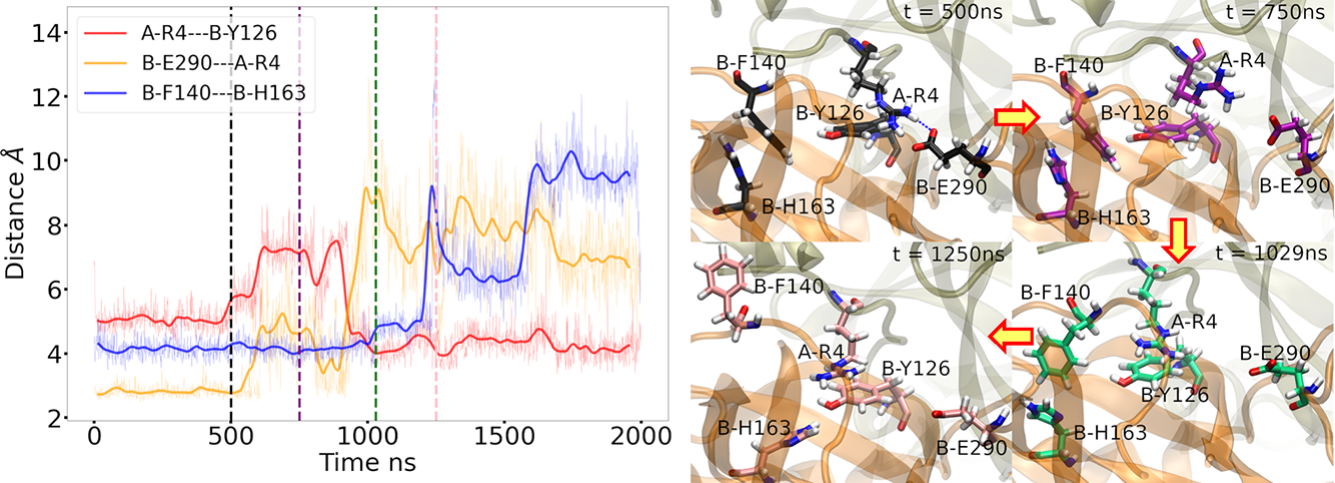
Symmetry-breaking of MPro coupled to the fatal breaking of salt bridge between A-Arg4 and B-Glu290 is observed in DESRES simulation. (a) Distances of different residual couples, namely, A-Arg4 and B-Tyr126, B-Glu290 and A-Arg4, B-Phe140 and B-His163. The black, purple, green, and pink vertical dashed lines stand for representatives at *t* = 500, 750, 1029, and 1250 ns. (b) Black, purple, green, and pink licorice structures of His163, Phe140, Arg4, Tyr126, and Glu290 in two subunits at *t* = 500, 750, 1029, and 1250 ns, respectively. The subunit A and B are represented by the tan-colored and orange-colored cartoon structure, respectively, and the blue dashed line represents the H-bonds.

Shaw et al.^60^ performed a 100-μs-long molecular dynamics simulation of MPro. The simulation revealed that there is asymmetrical behavior between the salt bridges of A/B-Glu290 and B/A-Arg4, which causes a cascade effect that disrupts the aromatic interactions among His163, Phe140, and His172. Figures 9 and S9 show that the salt bridges between A/B-Glu290 and B/A-Arg4 were destabilized after around 500 ns, increasing the mobility of both A/B-Arg4 residues in the surrounding water. This is demonstrated by the significant increase in the distances between A/B-Arg4 and B/A-Tyr126 around *t* = 750 ns. At *t* = 1029 ns, A-Arg4 formed a stable cation−π interaction with B-Tyr126, reducing the distance between them to about 4 Å (Figure 9). Interestingly, the distances between A-Arg4 and B-Glu290 showed an opposite trend, suggesting a sequential replacement of the interactions from salt bridges to cation-π stacks in subunit B. In subunit A, the distances of A-Glu290---B-Arg4 and B-Arg4---A-Tyr126 exhibited the same trend, increasing dramatically since *t* = 500 ns and remaining stable at high distances >6 Å until about 1000 ns (Figure S10). The stable cation−π interaction between A-Arg4 and B-Tyr126 broke the hydrophobic interaction between B-Phe140 and B-Tyr126, which destabilized the aromatic system, including B-Phe140, B-His163, and B-His172 at around *t* = 1250 ns (Figure 9). However, B-Arg4 occasionally formed a salt-bridge connection with A-Glu290 after a period of freely fluctuating, as indicated by the drop in distance to around 4 Å^61^ (Figure S10).

## Discussion

### Levels of Consistency between Catalytic Site Features Are Supported by Crystal Structures

The high Tanimoto coefficient between hydrophobic packing between Phe140 and His163 and a “good” oxyanion hole (Figure 3) is supported by the observation that the features occur concurrently (either formed or broken) in all existing crystal structures of MPro. In contrast, our observation that the distance between Glu166 and His172 *can* behave independently is supported by an asymmetric holo-dimeric structure of MPro (PDB entry 6Y2G) solved by Zhang et al.^34^ Based on the distance between Glu166 and His172, they described one subunit as active and the other as inactive. However, a ligand is bound to *both* sites, and the oxyanion hole is preserved, demonstrating that the features can formed independently.

The π stack between Phe140 and His163 is a useful proxy for the oxyanion hole because the metric does not require the presence of a substrate. According to Chen et al.,^8^ oxyanion hole quality was assessed based on the distance between a Gln residue *in the substrate* and the amide groups of Gly143, Ser144, and Cys145.^8,41^ By these metrics, determination of whether an oxyanion hole is considered “good” or “not good” necessitates the presence of a bound substrate.

### Our Analysis Disfavors the Half-of-Sites Activity Hypothesis But Does Not Completely Rule It Out

The title of an influential paper by Chen et al.^8^ proclaims that even though SARS-CoV MPro is a dimer, only one protomer of MPro is active at a time. This caused our team great confusion, as we thought that we would observe this behavior in the Folding@Home simulations. Our analysis of the Folding@Home simulations contradicts this hypothesis, as it suggests that both subunits can assume an active conformation at the same time, and indeed that the 1–1 state is much more highly populated than either the 1–0 or 0–1 state (Figure 4).

On the other hand, the hypothesis is supported by shorter molecular dynamics simulations. The original basis of the hypothesis was a total of 148 ns of molecular dynamics (a reasonable amount given the computing power available at the time in 2006), in which Chen et al.^8^ observed unidirectional transitions from symmetric and active conformations of SARS-CoV MPro to conformations with a single inactive protomer. Similar behavior is observed in the 100 μs DESRES simulation of the SARS-CoV-2 MPro. As the Folding@Home simulations include more than 2 ms of data and sample many more transitions, they are more statistically reliable. However, it is unclear whether the force field used by DESRES or Folding@Home is more accurate. Thus, the hypothesis should be evaluated in the context of other data.

Other data reported by Chen et al.^8^ do not conclusively support half-of-sites activity over a simpler hypothesis. In addition to the simulations, the authors also reported the activity of MPro as a function of the wild-type enzyme concentration (Figure 2 in their study). Consistent with the importance of dimerization, activity increases quadratically with concentration. They also performed a clever experiment in which the wild-type enzyme concentration was increased in the presence of excess inactive C145A mutant. They found that activity increases linearly with concentration and that the hybrid dimer has about one-half the activity of the wild-type dimer. As they concluded, these results show that only one active protomer is required for the activity of the dimer. However, the experiment does not show that in the wild-type dimer, only one protomer is active at a time. They argued that in the wild type, one protomer is active and the other inactive, whereas in the hybrid, the wild-type protomer alternates between active and inactive forms. However, their enzyme activity data can also be explained by a simpler model in which wild-type protomers are primarily active, whether as part of a wild-type or hybrid dimer.

Crystallographic evidence supports the ability of MPro to exist in the 1–1 state. In a majority of MPro crystals, both subunits are in the active state. The physiological relevance of this characteristic may be debated because most of these structures were determined in space group C2 and comprise only one subunit in the asymmetric unit. However, Shaqra et al.^6^ recently solved SARS-CoV-2 MPro structures with a dimer in the asymmetric unit in which both active sites are simultaneously bound to *substrates*. This does not completely rule out the possibility that even though both sites can bind substrates, only one of the sites can perform catalysis.

The strongest evidence opposing the half-of-the-sites activity hypothesis are studies that vary pH. Yang et al.^16^ solved crystal structures of SARS-CoV MPro at pH 6.0, 7.6, and 8.0 and measured enzyme activity at several pH between about 5.5 and 8.5. In the crystal structures, one protomer appears active at all pH values, but the other protomer appears inactive at pH 6.0. Activity is also about half the maximum at pH 6.0. The combination of crystallography and enzyme activity suggests that at pH 6.0, the dimer has an *average* of one active and one inactive subunit. However, the fact that MPro *can* have one active and inactive subunit at certain pH does not imply that only one subunit can be active at a time at any pH. Indeed, it suggests that at pH levels with maximal activity both subunits are active, contradicting the half-of-sites activity hypothesis. Comparable pH activity profiles have been observed in other studies with SARS-CoV MPro^61,62,63^ and SARS-CoV-2 MPro.^17^

The half-of-sites activity hypothesis cannot be completely ruled out due to the possibility that the half-active structure at pH 6.0 is a crystallographic artifact. As with Yang et al.,^16^ Tan et al.^63^ solved half-active structures of SARS-CoV MPro at pH 6.0. However, they also solved structures at pH 5.9 and 6.6 in which both subunits are inactive. The determination of a variety of conformational states for the same protein construct by the same group in the same article demonstrates the sensitivity of the catalytic site conformations to buffer conditions and crystallographic contacts.

### Inactive Conformations in Folding@Home Simulations Are Consistent with Previous Simulations Modeling Low pH

Based on constant pH molecular dynamics simulations, Verma et al.^28^ estimated that in SARS-CoV-2 MPro, His172 has a p*K*_a_ of 6.6 in both subunits. For SARS-CoV MPro, His172 was estimated to have a p*K*_a_ value of 6.6 in one subunit and 7.0 in the other. Thus, they proposed that H172 is responsible for the pH activity switch observed by Yang et al.^16^ Subsequently, they performed 2 μs of fixed-charge molecular dynamics simulations with a positive charge on His172. They observed that His172 forms a salt bridge with Glu166 and that Phe140 rotates away from His163. In the inactive conformation of protomer A, His172 and Glu166 come into close proximity, and Phe140 also rotates away from His163 (Figure 6). This suggests that low pH and the protonation of His172 can stabilize an inactive conformation that exists (with low probability) at neutral pH.

Enzymatic activity assays of His172 mutants corroborate the role of the residue as a pH-dependent activity switch.^17^ At pH 7.0, the mutant with tyrosine, which has steric and electrostatic properties similar to those of neutral histidine, maintains activity comparable to that of the wild type. The alanine mutant is also neutral but cannot replace the structural role of the histidine side chain, leading to a significantly reduced activity. In both mutants, the pH profile has a lower p*K*_a_ than the wild type—close to 6 opposed to 6.7—suggesting that another residue assumes the role of the activity switch. Finally, like histidine would be at low pH, lysine and arginine are positively charged and likely to inactivate the enzyme in a similar way.

### Mutagenesis Studies Suggest that Some Residues with High JSD between Active and Inactive Conformations Have an Important Structural Role

Flynn et al.^64^ mapped a comprehensive fitness landscape of SARS-CoV-2 MPro by performing a mutational scan of every position to all other amino acids and evaluating their function in yeast. The only Phe3 mutants retaining over half the activity were the hydrophobic residues tryptophan, leucine, methionine, and isoleucine. Previously, it was shown that the F3A mutant of SARS-CoV MPro has a similarly dramatic effect on activity but minimal effect on dimerization.^65^ Our analysis suggests that mutations that eliminate the hydrophobic interaction between Phe3 and the L1 loop (Figure 8) destabilize the active conformation and shift the conformational equilibrium toward an inactive form of the enzyme. For SARS-CoV MPro, dimerization affinity is weakened, and enzyme activity is reduced by R4A^65^ and even more so by the charge-flipping R4E.^25^ However, SARS-CoV-2 MPro tolerates a broad range of mutations at Arg4,^64^ suggesting that the salt bridge is not essential to enzyme function. Like Phe3, Tyr118 only tolerates mutation to a subset of hydrophobic residues.^64^ Our analysis suggests that the Y118F mutant, in particular, has diminished activity because it lacks a hydrogen bond that stabilizes the active conformation but can form the π stack with Phe140 seen in inactive conformations (Figure S8). Similarly, the F140Y mutant reduces activity because it introduces an alcohol group that is sterically occluded in active conformation but is tolerated, not interfering with the π stack with Tyr118 in the inactive conformation of subunit A (Figure S8). (It is less obvious how the mutation affects subunit B.) For unclear reasons, SARS-CoV-2 MPro only mildly tolerates the mutation of His163 to Glu. For His172, the fitness landscape in yeast^64^ suggests that phenylalanine can replace the π stack observed in active conformations and removes the hydrogen bond that favors inactive conformations. All mutations of Glu290 severely reduce the activity of SARS-CoV-2 MPro.^64^ For SARS-CoV MPro, the E290A mutant weakens dimerization (as with Arg4) and enzyme activity could not be measured.^66^ Together these results show that the Arg4-Glu290 salt bridge does affect dimerization but that Glu290 plays an additional essential role in enzymatic activity. As for Gln299, its importance in maintaining the active conformation is corroborated by the limited number of mutants that maintain appreciable activity in both SARS-CoV^9^ and SARS-CoV-2.^64^

### Consistency between Our Results and Experimental Studies Highlights the Power of Our Approach in Identifying Key Residues Involved in Structural Transitions

Compared to our analysis based on the JSD, the projections of RMSD values of residues’ torsion angles (Figure S7) between the crystal structures of 1–0 (PDB entry 1UJ1) and 1–1 (PDB entry 6Y84), or between 0–0 (PDB entry 7NIJ) and 1–1 (PDB entry 6Y84), reveal a higher number of residues undergoing conformational changes potentially involved in active–inactive transitions. This indicates that relying solely on the inactive conformations of SARS-CoV-2 MPro crystal structures is insufficient for gaining insight into interesting mechanisms. In contrast, our analysis has proven useful in identifying significant residues associated with inactivation.

## Conclusions

We provide an atomistic explanation for several crucial aspects of MPro inactivation. Through the integration of the MSM, TPT, and JSD, we have identified pivotal residues involved in transitions between active and inactive conformations. In active conformations, aromatic stacking interactions of residues Phe140, His163, and His172 play an important role in the stabilization of the oxyanion hole. Each subunit has a distinct inactive conformation characterized by π stacking interactions among Phe140, His163, His172, and Tyr118 coupled with a hydrogen bond network centered around His163 and His173. Subunit B is more frequently inactive than subunit A. Folding@Home simulations contradict the half-of-sites activity hypothesis for SARS-CoV-2 MPro, instead suggesting that both subunits can be active simultaneously. Our research not only enhances our understanding of SARS-CoV-2 MPro deactivation but also demonstrates how computational methods can contribute to enzymology.

## Data Availability

The Folding@Home and DESRES MPro simulations that we analyzed are available on MOLSSI (https://covid.molssi.org/simulations/). The custom scripts that we used to analyze the data and generate figures for this manuscript are available on GitHub (https://github.com/EllaNguyen1711/Mpro_inactivation). The package that we wrote to efficiently compute occupancy fingerprints is also available on GitHub (https://github.com/jimtufts/occupancy_fingerprinter).

## Supporting Information Available

Flux pathways, 1D energy landscapes, implied time scales, comparisons to crystal data, committor probabilities, supporting JSD and RMSD figures, probability distributions, and time series graphs (PDF)
