# Supplementary material for: On Inactivation of the Coronavirus Main Protease
Source: J Chem Inf Model. 2024 Feb 29;64(5):1644–56. doi: 10.1021/acs.jcim.3c01518 (PMC10936523; doi:10.1021/acs.jcim.3c01518)
Supplement: Supplementary file 1 — ci3c01518_si_001.pdf [file ci3c01518_si_001.pdf]

SUPPORTING INFORMATION

On Inactivation of the Coronavirus Main Protease

Thi Hong Ha Nguyen[1], James Tufts[1], David D. L. Minh*[1]

[1]Department of Chemistry, Illinois Institute of Technology, Chicago, IL 60616, USA

* To whom correspondence should be addressed:

David D. L. Minh: dminh@iit.edu

Table S1. Summary of histidine protonation states. If the neutral histidine was protonated at the epsilon position on the imidazole ring, it is listed as under the Epsilon column. If it is on the delta nitrogen, it is listed in the Delta column. The pH column lists experimental conditions under which the crystal structure was collected.

Trajectory	Epsilon	Delta	pH of Crystal Structure
Folding@Home	His41, His64, His163, His164, His172, His246	His80	7.0
D.E. Shaw Research	His41, His64, His163, His164, His172, His246	His80	6.5

Table S2. Pathways with the greatest flux, totaling over 95% of the total flux**.**

Path flux	Path % of total flux	Cumulative % of total flux	Paths
1.01E-04	50.442	50.442	['1-0' 'I1' 'I2' 'I3' '0-1']
3.31E-05	16.509	66.951	['1-0' 'I4' '0-1']
2.04E-05	10.138	77.089	['1-0' 'I2' '0-1']
2.02E-05	10.055	87.144	['1-0' 'I4' 'I3' '0-1']
1.42E-05	7.068	94.213	['1-0' 'I1' 'I3' '0-1']
3.49E-06	1.737	95.949	['1-0' 'I1' 'I4' 'I2' '0-1']

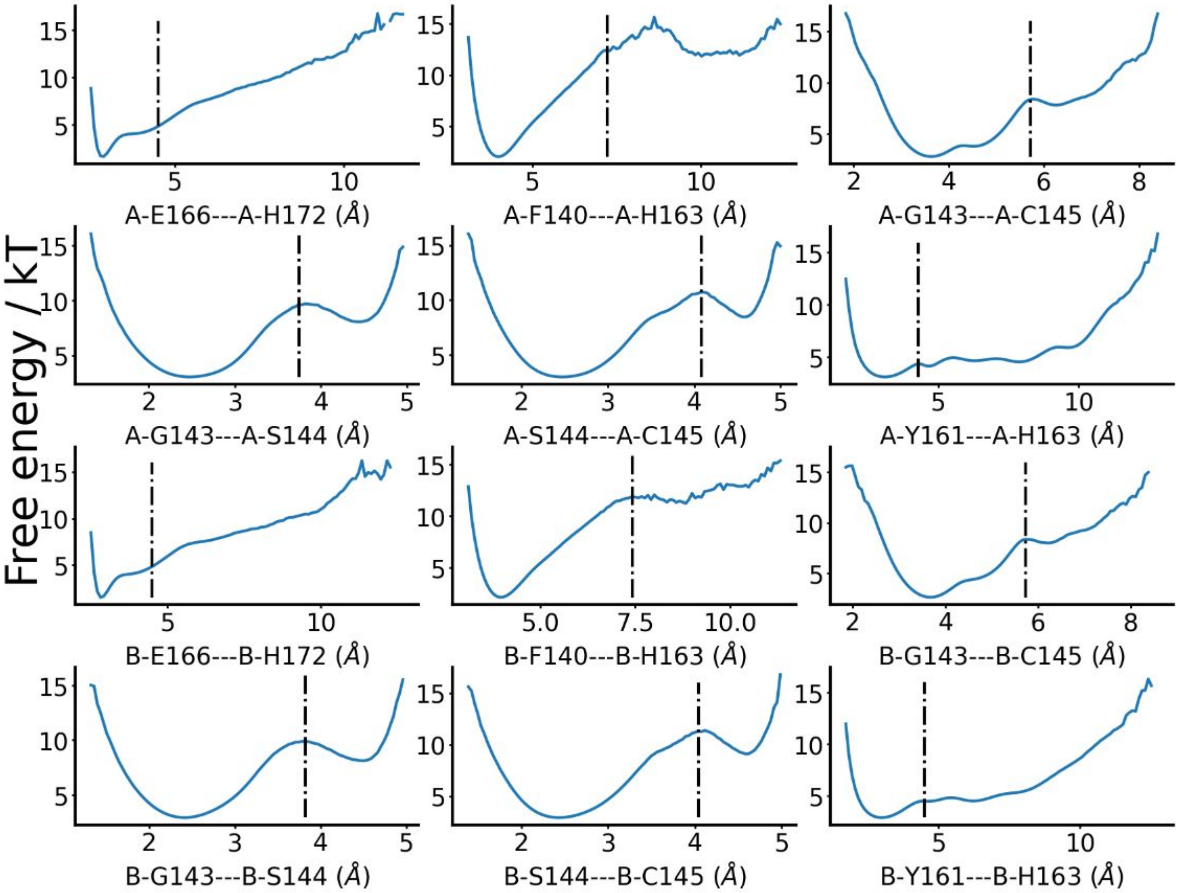

Figure S1. One-dimensional energy landscapes of MPro. Each is based on a different distance that describes one of four features used to define the active and inactive conformations of MPro. The thresholds are represented by the black dashed lines.

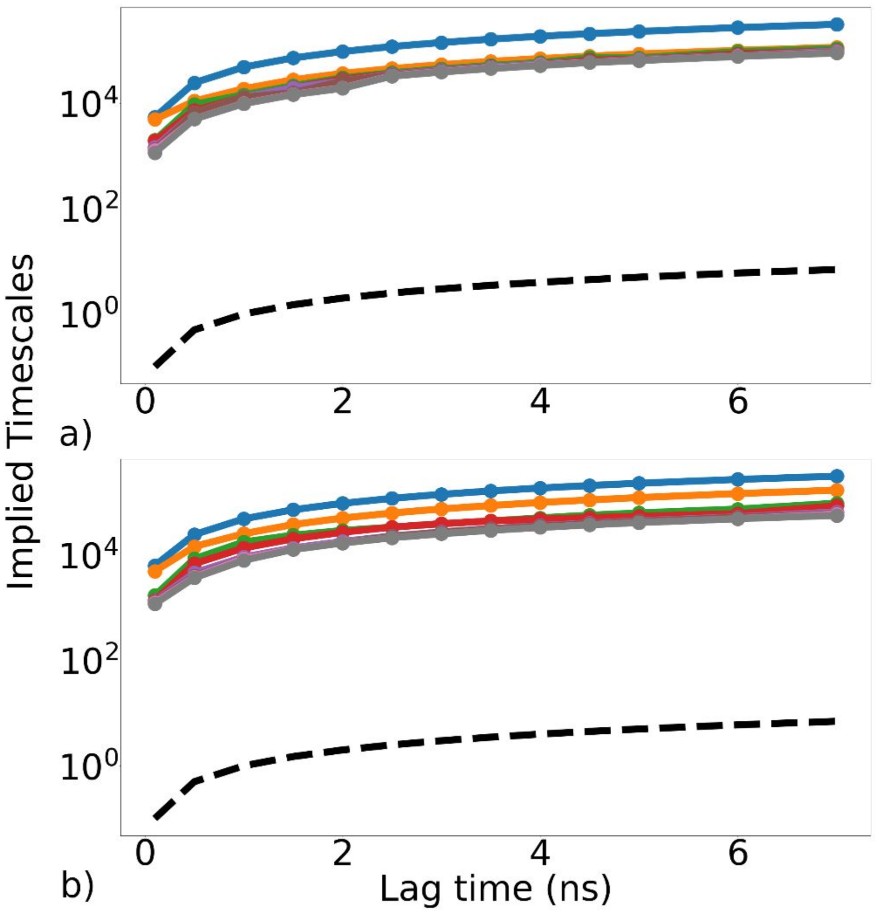

Figure S2. Implied timescales as a function of lag time for MSMs based on (a) single-layer and (b) two-layer clustering. The lag time is shown as a dashed line. Different colors represent different eigenvalues of the transition matrix.

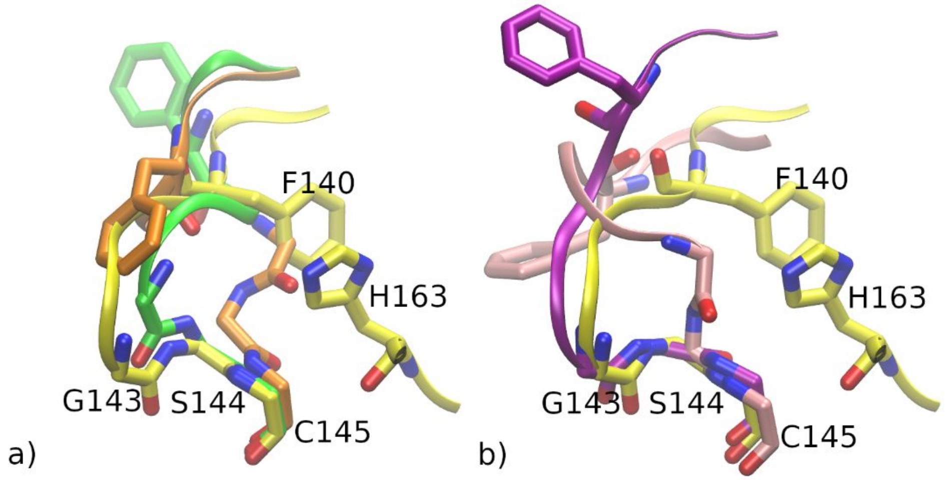

Figure S3. Comparison of aligned simulated and crystallographic structures of MPro. Both panels include an active conformation of MPro from PDB entry 6Y2E (yellow). a) Inactive crystallographic conformations of SARS-CoV-1 MPro in PDB entry 1UJ1 chain B (green) and of SARS-CoV-2 MPro in PDB entry 7NIJ chain A (orange). b) Comparable inactive simulated conformations from Folding@Home subunit A (pink) and DESRES subunit B (purple). The representative for Folding@Home was selected from microstates 0-1, while the one for DESRES was randomly chosen after the initial 1000 ns.

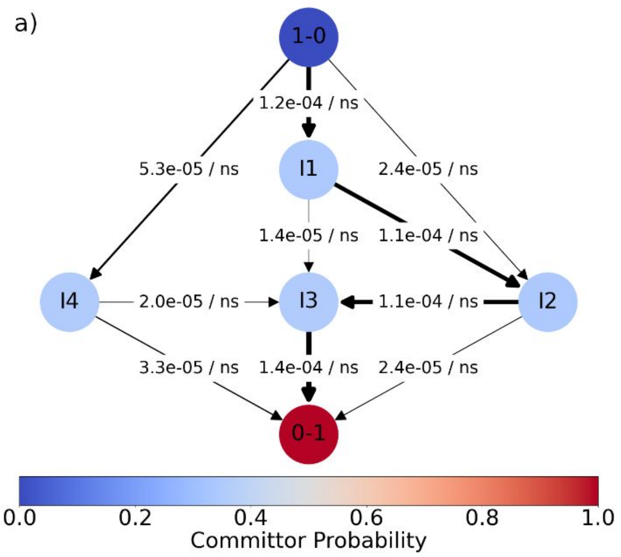

Figure S4. Committor probability and coarse-grained net flux of the most dominant pathways (totaling over 95% of the total flux). a) Transitions between 1-0 to 0-1 states. b) Transition between 0-1 to 1-0 states.

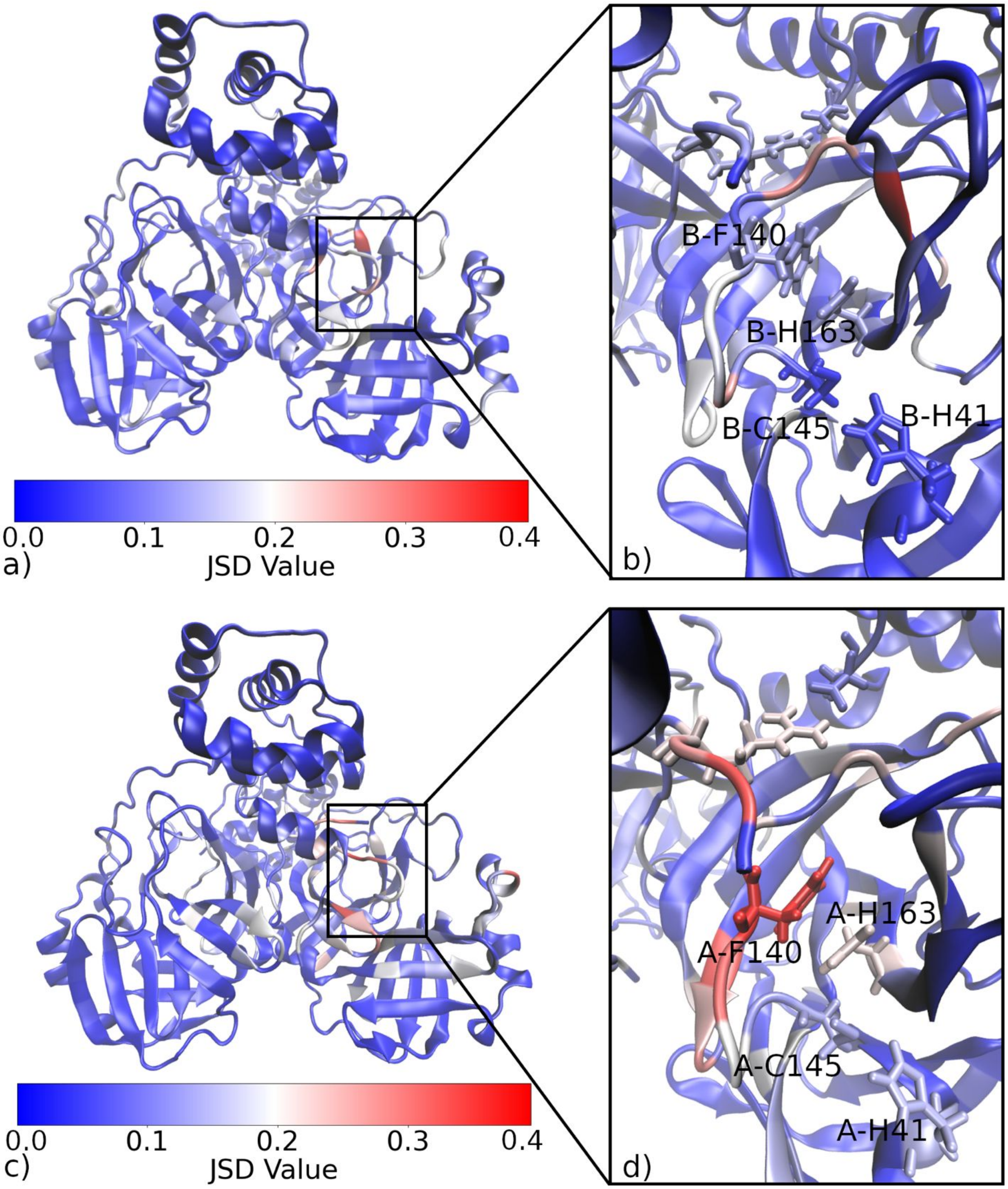

Figure S5. Projection of JSD values of residues over active-inactive transitions onto a crystal structure (PDB entry 6Y2E). (a, c) Full view of dimer colored by JSD values for the transition from I1 to 1-0 and from I3 to 0-1, respectively. (b, d) Zoom in of catalytic sites (a, c) which have highest JSD values in transitions of active-inactive of two subunits.

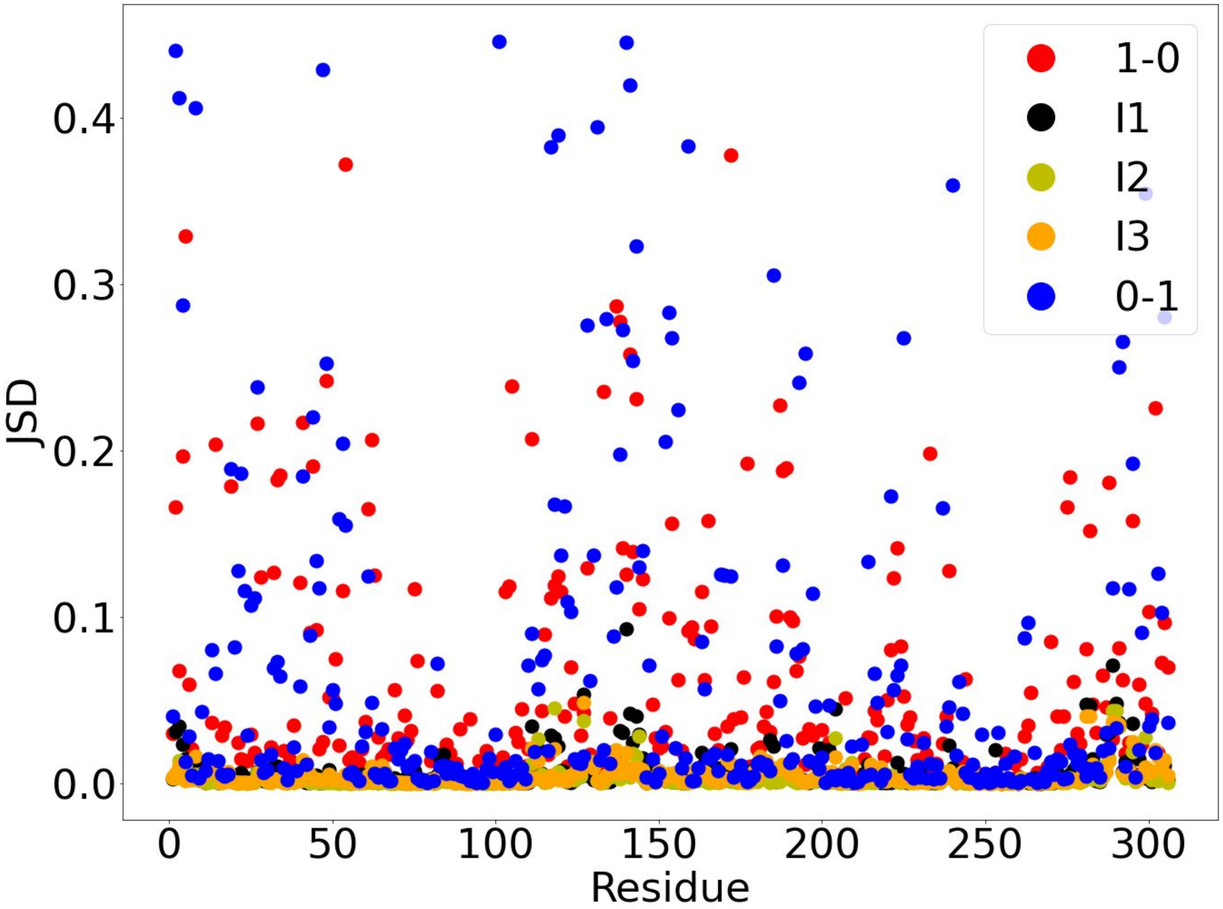

Figure S6. Jensen-Shannon Divergence (JSD) of residues' torsion angles between subunits A and B over macrostates. For each residue, the torsional angle with the maximal JSD value was used. Each residue has JSD values for five macrostates, which are colored red, black, yellow, orange, blue, respectively.

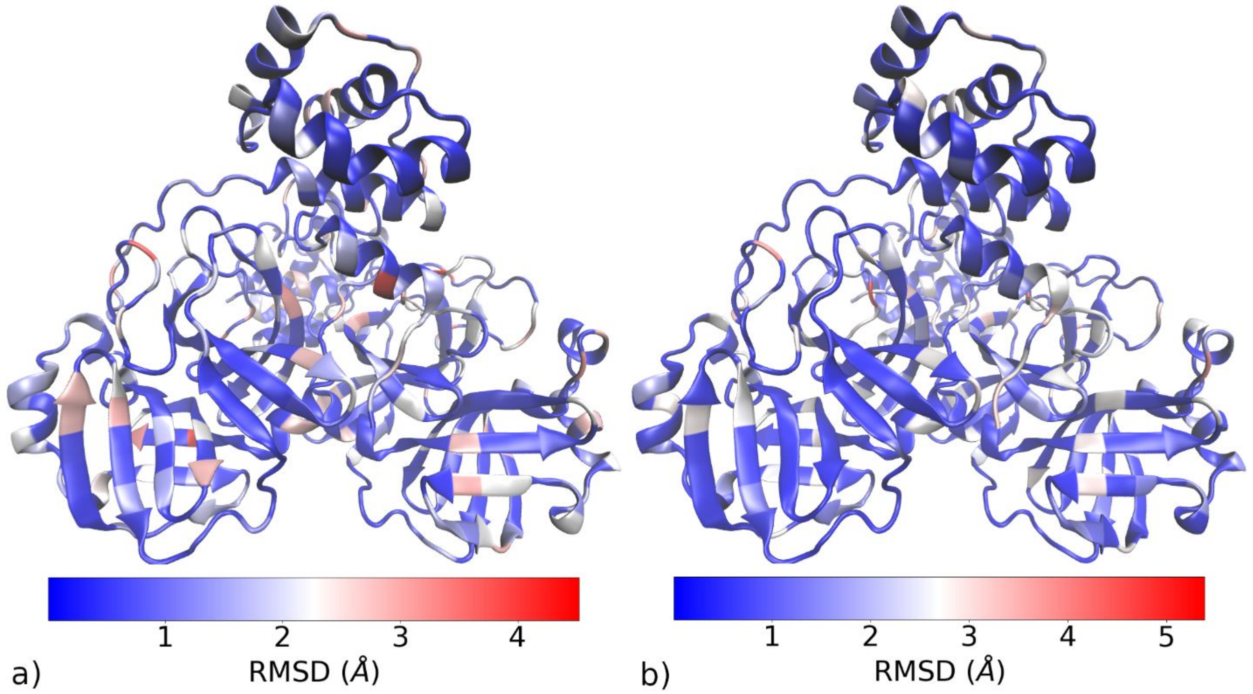

Figure S7. RMSD between inactive and active crystal structures. a) Projection of RMSD values of residues' torsion angles between an active-inactive crystal structure (PDB entry 1UJ1) and an active-active crystal structure (PDB entry 6Y84). b) Projection of RMSD values of residues' torsion angles between an inactive-inactive crystal structure (PDB entry 7NIJ) and an active-active crystal structure (PDB entry 6Y84).

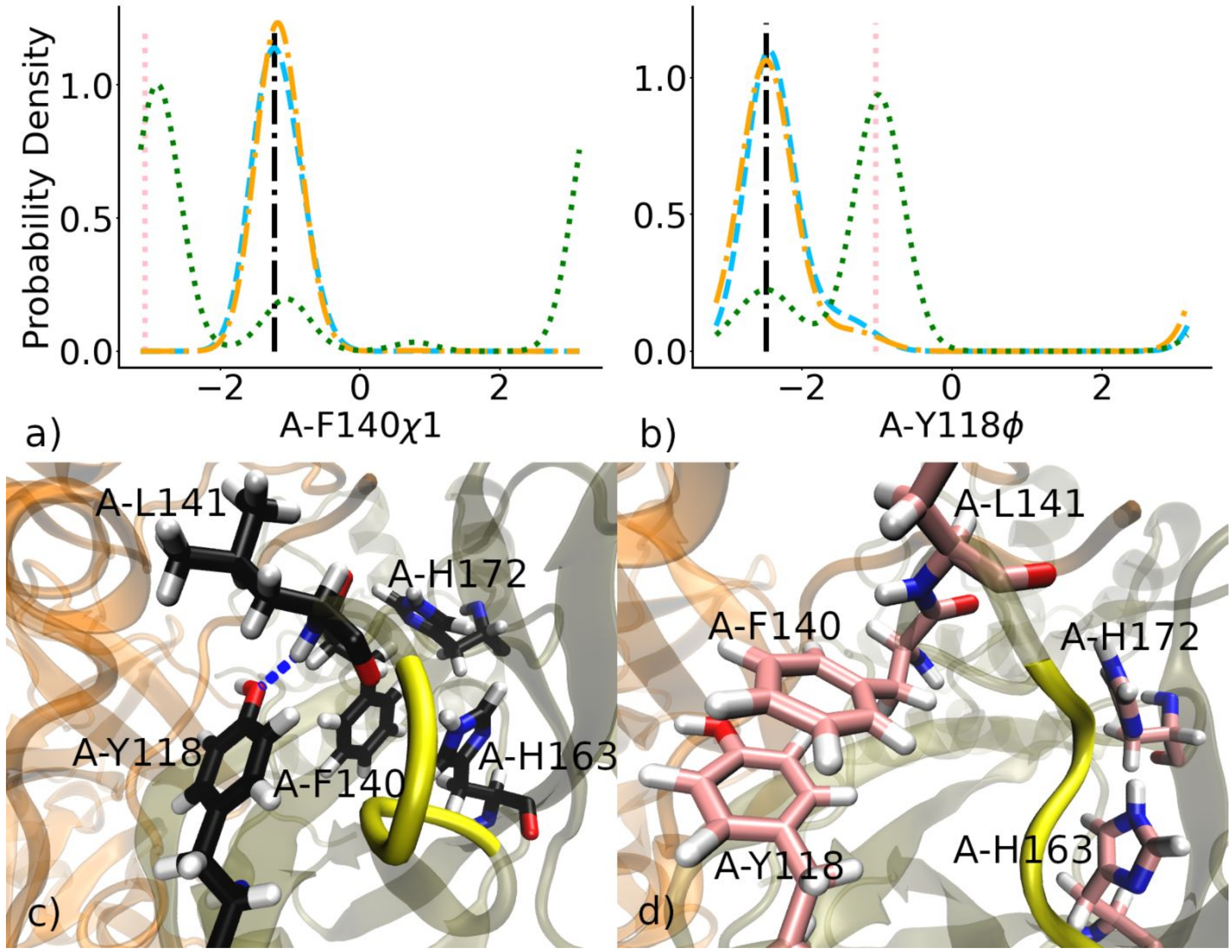

Figure S8. Inactivation of subunit A. (a,b) Probability distributions of the χ1 and Φ angles – which specify rotation of the sidechain and backbone of Phe140 and Tyr118, respectively, in subunit A for 1-0 (cyan dashed), 1-1 (orange dash-dotted), and 0-1 (green dotted) states. The 1-1 state is a weighted sum of I1 and I3. Angles observed in two of the representative structures (shown as a black cross and pink pentagon in Fig. 2) are shown as vertical lines of the corresponding colors. (c, d) These two representative structures are shown (coded by the color of the licorice) with the protein structure colored tan for subunit A and orange for subunit B. Blue dashed lines represent H-bonds.

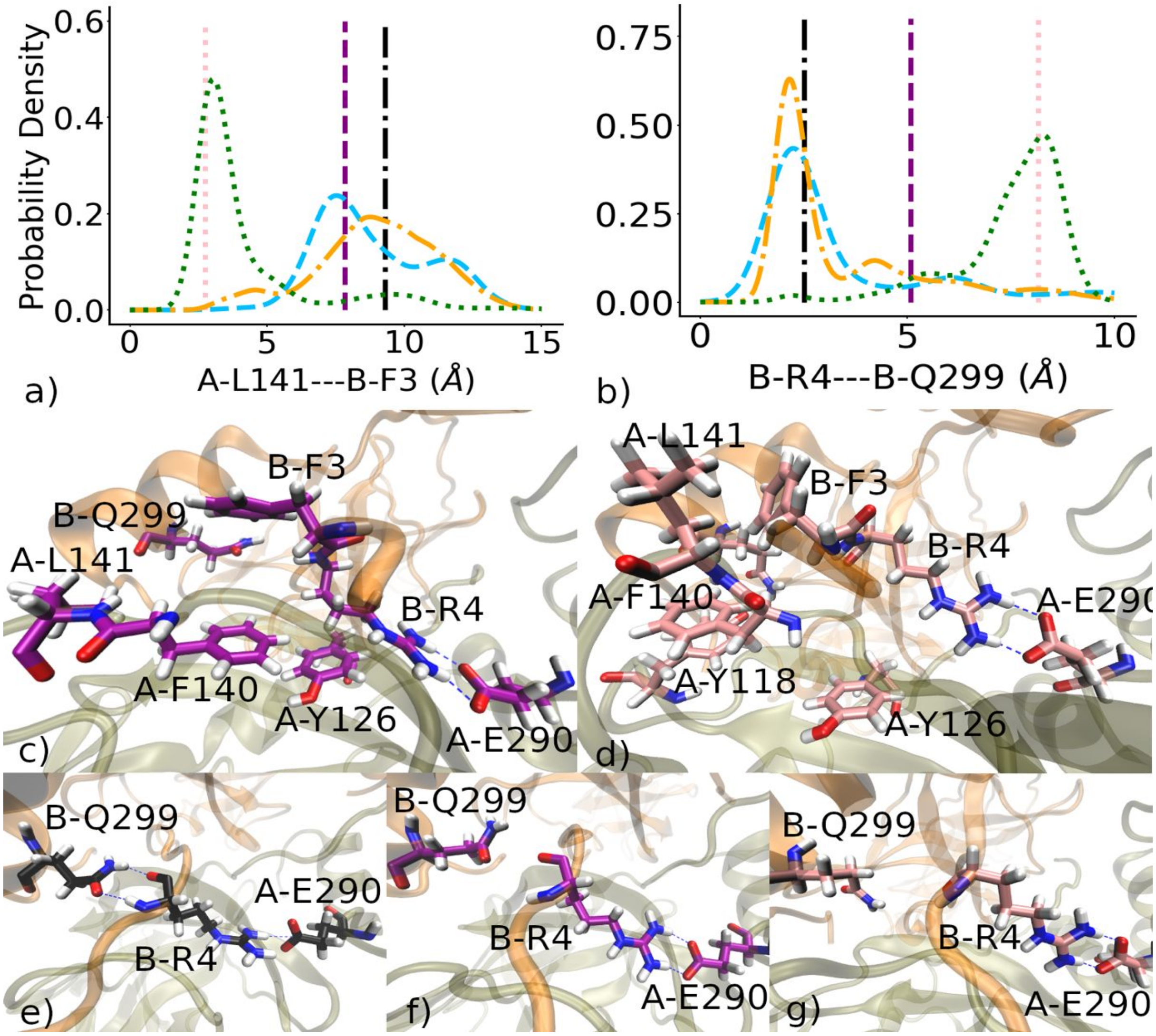

Figure S9. Replacement of pairing between specific residues in the dimer interface between the enzymatic site at subunit A and the N-terminus of subunit B. (a,b) Probability distributions of the distances, A-Leu141 and B-Phe3, B-Gln299 and B-Arg4, respectively, for 1-0 (cyan dashed), 1-1 (orange dash-dotted), and 0-1 (green dotted) states. The 1-1 state is a weighted sum of I1 and I3. Distances observed in three of the representative structures (shown as a black cross, purple star, and pink pentagon in Fig. 2) are shown as vertical lines of the corresponding colors. (c, d, e, f, g) These three representative structures are shown (coded by the color of the licorice) with the protein structure colored tan for subunit A and orange for subunit B. Blue dashed lines represent H-bonds.

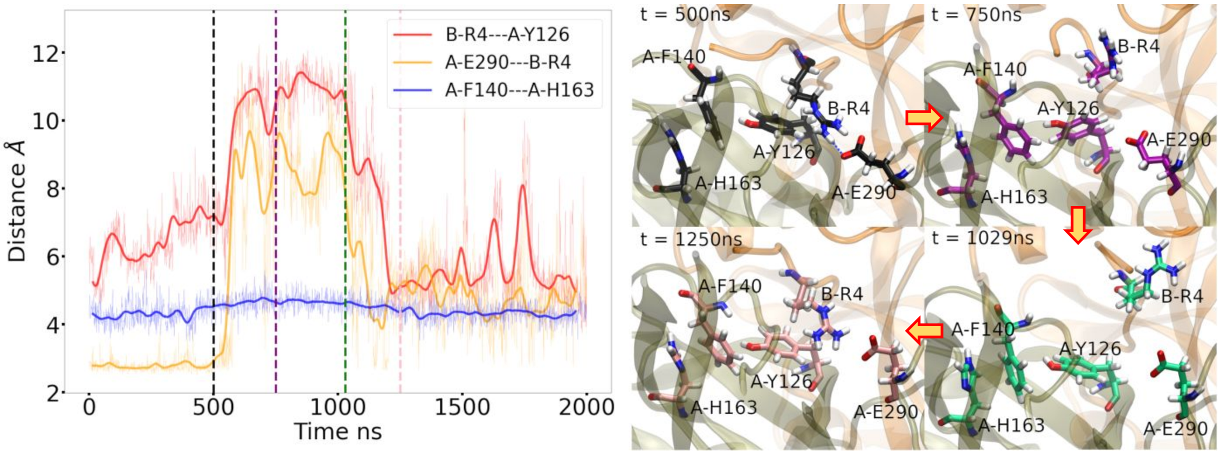

Figure S10. Symmetry-maintaining in subunit A of MPro after the breaking of salt-bridge B-Arg4 and A-Glu290 in DESRES simulation. a) Distances of different residual couples, namely B-Arg4 and A-Tyr126, A-Glu290 and B-Arg4, A-Phe140 and A-His163. The black, purple, green, pink vertical dash lines stand for representatives at t = 500, 750, 1029, 1250 ns. b) Black, purple, green, pink licorice structures of His163, Phe140, Arg4, Tyr126, Glu290 in two subunits at t= 500, 750, 1029, 1250 ns respectively. The subunit A and B are represented by the tan-colored and orange-colored cartoon structure, respectively, and the blue dashed line represents the H-bonds.
